# Advancing dengue vaccination using a T-cell priming peptide approach

**DOI:** 10.1016/j.ebiom.2024.105012

**Published:** 2024-02-14

**Authors:** Jéssica Pires Farias, Alexander Birbrair, Jaime Henrique Amorim

**Affiliations:** a Western Bahia Virology Institute, Center of Biological Sciences and Health, Federal University of Western Bahia, Barreiras, BA, Brazil; b Vaccine Development Laboratory, Department of Microbiology, Biomedical Sciences Institute, University of São Paulo, Sao Paulo, SP, Brazil; c Department of Dermatology, School of Medicine and Public Health, University of Wisconsin-Madison, Madison, WI, USA

The persistent battle against dengue has been characterized by the formidable challenges posed by the virus's complex serotypes and the concerning risks associated with antibody-dependent enhancement (ADE) of infection, a phenomenon observed in some vaccine candidates targeting the envelope glycoprotein.^1^ In contributing to the ongoing discourse on dengue vaccination strategies, Miauton and colleagues embarked on a pioneering effort to assess the safety and immunogenicity of a novel candidate: a CD8+ T-cell priming, gold nanoparticle (GNP)-based, multi-valent, synthetic peptide dengue vaccine (PepGNP-Dengue).^2^ This unique approach is designed to elicit protective cellular immunity without invoking antibody responses.

In their comprehensive evaluation, the authors reported a noteworthy absence of vaccine-related serious adverse events, affirming the safety profile of PepGNP-Dengue. However, a case of cutaneous reaction that required hospitalized management was reported. Considering the small sample size of this phase I trial, this finding is significant and should be considered regarding the safety profile of this vaccine candidate.

Notably, as anticipated, the vaccine did not stimulate anti-dengue virus (DENV) antibodies. Instead, it significantly induced an increase in dengue-specific CD8+ T cells and memory cell subsets, marking a substantial breakthrough in the immunization against dengue. The standout achievement of this study lies in the successful induction of virus-specific CD8^+^ T cells by PepGNP-Dengue, signifying a shift in the paradigm of dengue vaccine development historically focused on the induction of^3^^,^^4^ neutralizing antibodies. This finding is particularly significant in the context of the limitations posed by ADE-associated risks and serotype-specific challenges that have impeded progress in the field.

The authors argue persuasively that their results provide crucial proof of concept for a vaccine strategy focused on conferring protective cellular immunity independently of antibody responses. While the robust cellular responses observed in this study are encouraging, the study's limited sample size necessitates caution in generalizing the findings to larger populations. There is no clinical evidence that T cells alone can control DENV infection, and prevent disease. Furthermore, the absence of assessments on interferon-gamma production and cytotoxic activity of CD8+ T cells constitutes notable limitations that warrant thoughtful consideration in the context of future research endeavors. Nevertheless, such a vaccine could be useful when applied with other vaccines to better elicit cellular immunity.

The exploration of dengue-specific T-cell epitopes as vaccine antigens represents a commendable and strategic approach. These epitopes, demonstrated to be conserved across dengue and other flaviviruses,^5^^,^^6^ open a promising avenue for addressing a broader spectrum of viral threats. Beyond dengue, the potential application of T-cell priming peptide vaccines targeting conserved epitopes could extend to other flaviviruses of significant epidemiological impact, such as *Zika virus*, *Yellow fever virus*, and *Japanese encephalitis virus*. Additionally, the conserved nature of T-cell epitopes in SARS-CoV-2^7^ suggests the possibility of designing a novel COVID-19 vaccine that could effectively control variants of concern, primarily selected based on epitopes for neutralizing antibodies present on the structural spike protein.^8^^,^^9^ Importantly, the reduced likelihood of ADE induction by vaccine antigens based on T-cell epitopes underscores the safety advantages of this approach compared to traditional antibody-focused strategies.

While Miauton and colleagues have undeniably made significant strides in demonstrating the feasibility of T-cell priming for dengue vaccination,^2^ the path forward necessitates more comprehensive investigations. Larger sample sizes and further exploration of T-cell functionality, including interferon-gamma production and cytotoxicity, are imperative to not only validate but also refine the observed immunogenicity. In future studies, a shift in the immunoproteomics approach to selecting epitopes based on experimental validation as lymphocyte targets, known antiviral cytokine profiles, and a demonstrated role in effective control of viral replication would provide a more targeted and informed strategy for inducing protective cellular immunity against dengue. The integration of such insights would undoubtedly enhance the translational potential of T-cell priming as a novel and promising avenue for dengue vaccine development, paving the way for more efficacious and safer immunization strategies in the ongoing global effort to combat dengue.

Furthermore, the future of dengue vaccine development should embrace the concept of immune protection through the death of infected cells induced by CD8^+^ T-cells. Understanding and harnessing this mechanism may provide a more effective means of eliminating virus-infected cells, thereby mitigating the risks associated with antibody-dependent enhancement. Additionally, the incorporation of superior adjuvants and advanced delivery systems for antigens holds promise in enhancing the induction of a robust protective immune response. Optimizing these components can potentially amplify the effectiveness of T-cell-mediated immunity, offering a more comprehensive defense against dengue infection.

In the ongoing quest for a dengue vaccine that is not only safe but also capable of conferring long-lasting protection, the integration of these future directions into research endeavors will be pivotal. By leveraging T-cell epitopes, understanding the nuances of CD8^+^ T-cell-mediated immunity, and refining the vaccine formulation with improved adjuvants and delivery systems, we can envisage a future where dengue vaccination becomes a more potent tool in our arsenal against the disease. These advancements will not only contribute to dengue prevention but may also pave the way for a broader, adaptable approach to combatting a range of viral infections.

## Declaration of interests

The authors declare no conflict of interest.
